# Eso-Sponge® for anastomotic leakage after oesophageal resection or perforation: outcomes from a national, prospective multicentre registry

**DOI:** 10.1093/bjsopen/zrac030

**Published:** 2022-04-22

**Authors:** Florian Richter, Alexander Hendricks, Bodo Schniewind, Jochen Hampe, Nils Heits, Witigo von Schönfels, Benedikt Reichert, Katrin Eberle, Mark Ellrichmann, Petra Baumann, Jan-Hendrik Egberts, Thomas Becker, Clemens Schafmayer

**Affiliations:** 1 Department of General, Visceral-, Thoracic-, Transplantation- and Paediatric Surgery, University Medical Center Schleswig-Holstein (UKSH), Campus Kiel, Kiel, Germany; 2 Department of General Surgery, University Hospital Rostock, Rostock, Germany; 3 Department of General Surgery and Thoracic Surgery, Hospital of Lueneburg, Lueneburg, Germany; 4 Medical Department I, University Hospital Dresden, TU Dresden, Dresden, Germany; 5 Department of Internal Medicine, Gastroenterology, Sophien-u. Hufeland Hospital, Weimar, Germany; 6 Department of Internal Medicine I, University Medical Centre Schleswig-Holstein (UKSH), Campus Kiel, Kiel, Germany; 7 Aesculap AG, Medical Scientific Affairs, Tuttlingen, Germany; 8 Department of Surgery, Israelitisches Krankenhaus, Hamburg, Germany

## Abstract

**Background:**

Anastomotic leakage (AL) after oesophagectomy and oesophageal perforations are associated with significant morbidity and mortality. Minimally invasive endoscopy is often used as first-line treatment, particularly endoluminal vacuum therapy (EVT). The aim was to assess the performance of the first commercially available endoluminal vacuum device (Eso-Sponge®) in the management of AL and perforation of the upper gastrointestinal tract (GIT).

**Methods:**

The Eso-Sponge® registry was designed in 2014 as a prospective, observational, national, multicentre registry. Patients were recruited with either AL or perforation within the upper GIT. Data were collected with a standardized form and transferred into a web-based platform. Twenty hospitals were enrolled at the beginning of the study (registration number NCT02662777; http://www.clinicaltrials.gov). The primary endpoint was successful closure of the oesophageal defect.

**Results:**

Eleven out of 20 centres recruited patients. A total of 102 patients were included in this interim analysis; 69 patients with AL and 33 with a perforation were treated by EVT. In the AL group, a closure of 91 per cent was observed and 76 per cent was observed in the perforation group. The occurrence of mediastinitis (*P* = 0.002) and the location of the defect (*P* = 0.008) were identified as significant predictors of defect closure.

**Conclusions:**

The Eso-Sponge® registry offers the opportunity to collate data on EVT with a uniform, commercially available product to improve standardization. Our data show that EVT with the Eso-Sponge® is an option for the management of AL and perforation within the upper GIT.

## Teaser

We present results of 102 patients recruited in a multicentre study assessing the performance of the first commercially available endoluminal vacuum device (Eso-Sponge®) in various indications (anastomotic insufficiency and perforation within the upper gastrointestinal tract). It seems to offer a safe, effective, and minimally invasive therapeutic approach with low mortality.

## Introduction

The incidence of oesophageal carcinoma is increasing and the prognosis is poor, with a median survival of less than 2 years and long-term survival rates below 15 per cent^1^. Despite continuous innovations of surgical treatment, oesophagectomy is a highly complex procedure with comparatively significant rates of postoperative complications. The incidence of anastomotic leakage (AL) secondary to oesophagectomy or gastrectomy ranges from 5 to 30 per cent and is associated with a morbidity and mortality rate of 20–50 per cent^2,3^. AL is defined as a defect of the intestinal wall at the anastomotic site with communication between intra- and extraluminal compartments^4^.

Oesophageal perforation after trauma or iatrogenic injury can lead to a life-threatening situation with mortality rates of up to 20 per cent^5,6^. Perforation is defined as a defect of the oesophageal wall that is caused iatrogenically, traumatically, or due to Boerhaave syndrome^7^. In addition to the size and location of the defect, clinical outcome is significantly influenced by the general condition of the patient^8,9^.

Timely and appropriate treatment is crucial in the management of oesophageal injuries. Over time, management has shifted from radical surgical intervention to more conservative measures, including endoscopic interventions. Treatment options include conservative treatment for small leak, surgical debridement, closure of the defect, or revising the anastomosis with simultaneous drainage^10^. Procedures such as endoscopic closure with clips, injection of fibrin glue, endoluminal drainage through gastric probes, endoluminal sutures, and the installation of endoscopic stent systems are also options^11,12^.

Endoluminal vacuum therapy (EVT) for treating AL and perforations has been successfully established and is increasingly used. Several studies have demonstrated high closure rates of approximately 90 per cent with a mortality rate of 10 per cent^13–16^.

In 2014 the European Society of Gastrointestinal Endoscopy (ESGE) suggested using endoscopy as a first-line intervention in leakages of the upper gastrointestinal tract (GIT)^17^.

To date, several low-volume single-centre studies with varying methodology have been published^15^.

The first commercially available endoluminal vacuum sponge system (Eso-Sponge®) of B. Braun for the endoscopic treatment of transmural defects within the upper GIT has been available since July 2014. It is CE marked and implemented in many centres for the treatment of perforations and ALs. A multicentre, prospective, web-based online registry was initiated in 2015 to evaluate the performance of the Eso-Sponge® in the upper GIT.

The aim of this study is to present interim results of a standardized multicentre registry of Eso-Sponge® therapy.

## Methods

### Patient recruitment and study design

The study design was a prospective, national, multicentre, open registry. The registry was sponsored and funded by B. Braun, Aesculap AG, Tuttlingen, Germany. The Medical Scientific Affairs department of Aesculap AG was responsible for project management, data management, statistics, and study registration (registration number NCT02662777; http://www.clinicaltrials.gov).

The objective of the present research was to evaluate Eso-Sponge® as an E-VAC therapy for AL and perforations within the upper GIT. Patients presenting with clinical suspicion of AL after oesophagectomy, such as elevated levels of C-reactive protein and pathological drainage fluid, had confirmatory endoscopic examination. Clinical impression and suspicious drain contents triggered radiological investigation with a CT thorax/abdomen.

An interim analysis of the first 100 patients to report results was planned.

Hospitals applying Eso-Sponge® in their daily clinical routine for E-VAC treatment in the upper GIT were eligible to participate. Twenty hospitals in Germany are participating and 11 centres are actively treating and including patients.

**Fig. 1:**
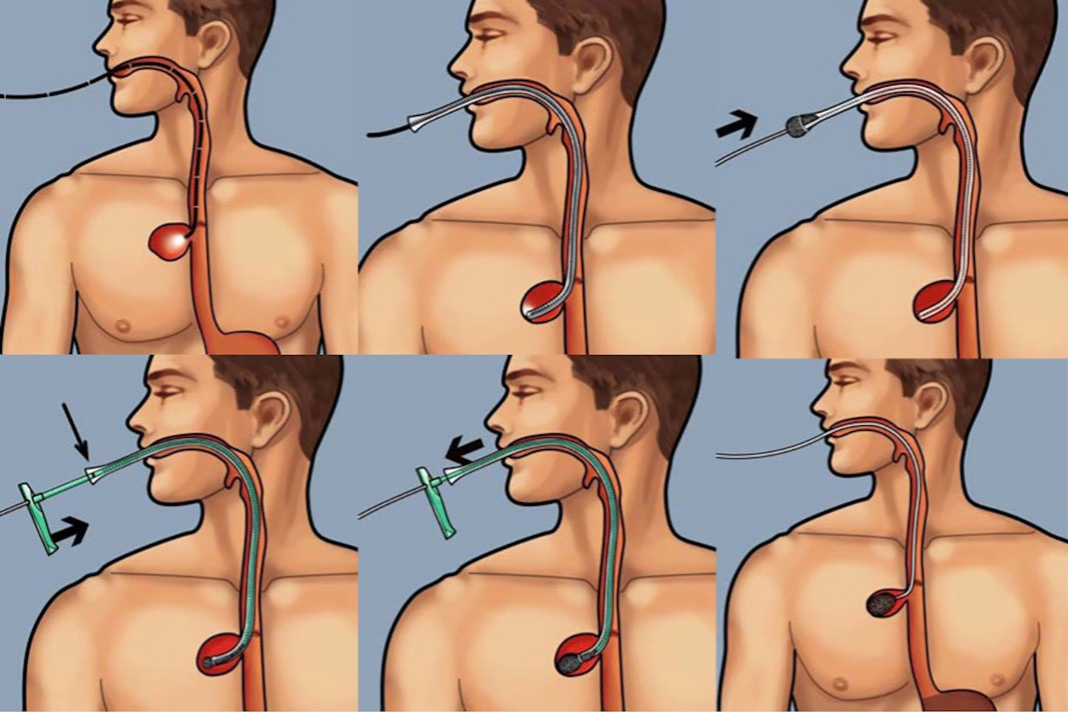
Endoscopic vacuum therapy

**Fig. 2:**
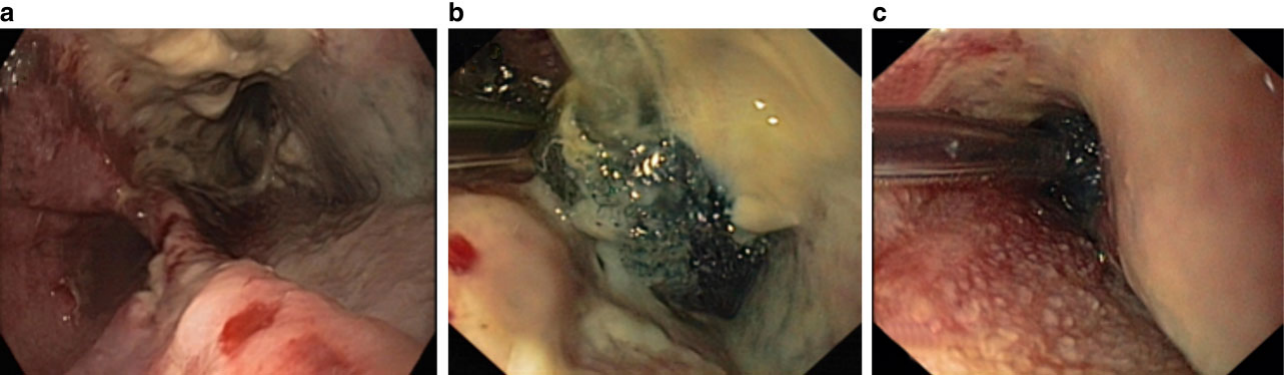
Endoscopic vacuum therapy

Patients were treated according to the local standards for E-VAC therapy and Eso-Sponge®, according to the manufacturer’s instructions. The Eso-Sponge® System (B. Braun Melsungen AG, Melsungen, Germany), is a minimally invasive method for E-VAC, consisting of a drainage tube with an attached open-pore sponge, a connection system, and an application system with a lavage set. *Fig. 1* displays an image of the Eso-Sponge®-procedure. The Eso-Sponge®-therapy was performed as described in previous publications^8^. Depending on the size of the defect, EVT was carried out either by endoscopic insertion of an Eso-Sponge® into the abscess cavity with an extraluminal placement, or in case of a limited defect size and the absence of a cavity, by endoscopic insertion in the lumen of the oesophagus itself as a luminal covering of the leak (*Fig. 2a–c*). To better compare the different oesophageal leakages, they were divided into three groups. We based this classification exclusively on the depth and/or diameter of the leakage. For defects, a diameter and/or depth of 0–1 cm was described as small, 1–4 cm was described as medium, and more than 4 cm was described as large. Over the course of treatment, with a reduction in defect size, the sponge placement could be moved from its initial intracavitary location to an intraluminal positioning allowing for complete closure of the leakage.

Controlled continuous negative pressure (100–125 mmHg) was applied via a transnasal gastric tube using an electronic vacuum pump (Eso-Sponge® by B.Braun, Aesculap AG, Tuttlingen, Germany)

**a**,**b** Endoscopic insertion of an Eso-Sponge® into the abscess cavity by means of an extra luminal placement. **c**, In cases of limited defect size and the absence of a cavity, endoscopic insertion of a sponge in the lumen of the oesophagus itself as a luminal covering of the leak.

The primary endpoint was the successful closure of the oesophageal defect, defined as the point when the defect size became too small for further sponge placements, and the surface was epithelialized. Data were collected on a case report form and transferred to a web-based platform offered by the sponsor (*Fig. S1*). *Table 1* displays the parameters used to describe the baseline, treatment details, outcomes, and factors influencing the clinical outcome. Patients were recruited consecutively, and informed consent was obtained before enrolment. The procedures were conducted in accordance with the ethical standards of the Committee on Human Experimentation of the institutions (A141/14).

**Table 1.** Parameters that define the baseline, treatment details, outcome of the treatment and to identify influencing factors of the Eso-Sponge® therapy success

Parameters to describe the baseline, the treatment details, and outcome of the treatment	Parameters to identify influencing factors of the ESO-Sponge® therapy success
Number of used sponges	Age
Mean replacement intervals	Sex
Duration of E-VAC treatment	Body mass index
Healing rate	Diabetes (insulin-dependent)
Complication rate	Smoking
Death rate	Alcohol consumption
Reoperation rate	Cancer diagnosis
Dysphagia score	Neoadjuvant therapy
	Anastomosis (*versus* perforation)
	Defect localization
	Stent applied
	Days to leakage diagnosis
	Distance from the arch (cm)
	Defect volume (log ml)

E-VAC, endoluminal vacuum.

### Evaluated variables

The evaluation protocol was divided into three main sections: the screening/initial data collection, E-VAC treatment, and follow-up. Complications (bleeding, fistula, abscess, peritonitis, pneumonia, mediastinitis, sepsis, renal failure, pleural empyema, and stenosis), mortality, and reoperations until the end of E-VAC treatment were entered into the web-based platform. The rate of granulated cavity, the number of endoscopic procedures, the total number of used sponges, the average replacement intervals, the duration of sponge treatment, and the dysphagia score after treatment were listed. Postinterventional ingestion discomfort was determined by a dysphagia score system, which was modified with the scoring method described by Mellow and Pinkas^18^.

### Statistics

All patients who had a surgical intervention with the product under investigation without any eligibility violation were included in the analysis.

Statistical analyses were performed for all subsets of clinical parameters. Kaplan–Meier survival estimation was used for calculation of complication rates if appropriate. For univariate analysis, the statistical significance was assessed by the log rank test. We used a Student’s *t* test and ANOVA for parametric data. Distribution and frequencies of categorical data were compared by Pearson’s chi-squared test. A *P* value < 0.05 was used for statistical tests to identify significance. Multivariate mixed effects logistic regression models were used to analyse predictors of response (granulated cavity). The backward elimination procedure with a ‘remain-in-model’ threshold of *P* < 0.10 was applied to detect covariates. Statistical calculation and testing were performed with SAS software version 9.4 (SAS, Cary, North Carolina, USA).

## Results

### Patient demographics and clinical characteristics

Patient recruitment started on 27 October 2014. A total of 102 patients had been recorded on the registry by 4 January 2019. In total, 69 (67.6 per cent) patients were diagnosed with an AL after oesophagectomy and 33 (32.4 per cent) with a perforation of the upper GIT.

The cohort comprised 72 (70.6 per cent) men and 30 (29.4 per cent) women. The mean patient age at the time of EVT was 60.9 years (23–82 years) in the AL group and 61.7 years (38–94 years) in the perforation group. Causes of iatrogenic perforation (27.3 per cent) were due to endoscopic procedures for biopsies or diagnostics, and fistula formation after chemoradiotherapy. The Boerhaave syndrome (21.2 per cent) was the most common cause of spontaneous perforation.

Oncological resection of distal oesophageal tumours was the most performed procedure (69.3 per cent), followed by bariatric procedures (11.8 per cent), and oncological surgery resecting the stomach (15.9 per cent) in the AL group. Histological examination demonstrated an adenocarcinoma in 73.9 per cent of cases, squamous cell carcinoma in 4.4 per cent, and no malignancy in 7.2 per cent of the subgroup. In these cases, the initial surgery was due to a non-malignant disease. Because of previous confirmatory histology/emergency presentations, histological specimen collection was not performed in 34.3 per cent of the patients. A full synopsis of the clinical data for each subgroup is given in *Tables 2* and *3*.

**Table 2.** Patient and treatment characteristics of the anastomotic leakage group

	Anastomotic leakage *n* (%)
	69 (100)
**Age (years)**	
Under 70	53 (76.9)
70 or older	16 (23.1)
**Sex**	
Male	53 (76.9)
Female	16 (23.1)
**Diagnosis**	
Oesophageal cancer	44 (63.8)
Obesity	8 (11.6)
Cancer of the gastric cardia	8 (11.6)
Other	9 (13)
**Histology**	
Adenocarcinoma	51 (73.9)
Squamous cell carcinoma	3 (4.4)
No malignancy	5 (7.2)
Not done	10 (14.5)
**Previous treatment**	
Chemoradiotherapy	7 (10.1)
Chemotherapy	32 (46.4)
None	30 (43.5)
**Resection type**	
Oesophagectomy	48 (69.3)
Endoscopic procedure	1 (1.5)
Gastrectomy	11 (15.9)
Gastric sleeve resection	3 (4.5)
Gastric bypass	5 (7.3)
Others	1 (1.5)
**Reconstruction type**	
Oesophagogastrostomy	53 (76.9)
Oesophagojejunostomy	6 (8.7)
Gastrojejunostomy	6 (8.7)
No reconstruction	4 (5.7)
**Site of reconstruction**	
Intrathoracic	49 (71.1)
Abdominal	15 (21.7)
Thoracoabdominal	1 (1.5)
No reconstruction	4 (5.7)

**Table 3.** Patient and treatment characteristics of the perforation group

	Perforation *n* (%)
	33 (100)
**Age (years)**	
Under 70	24 (72.7)
70 or older	9 (27.3)
**Sex**	
Male	19 (57.6)
Female	14 (42.4)
**Diagnosis**	
Oesophageal cancer-lower	1 (3.1)
Obesity	3 (9.1)
Iatrogenic perforation	9 (27.3)
Boerhaave’s syndrome	7 (21.2)
Other	13 (39.3)
**Histology**	
Squamous cell carcinoma	3 (9.1)
No malignancy	5 (13.4)
Not done	25 (77.5)
**Previous treatment**	
Chemoradiotherapy	3 (9.1)
None	30 (90.9)
**Resection type**	
Oesophagectomy	3 (9)
Endoscopic procedure	14 (42.5)
Gastrectomy	2 (6)
Gastric sleeve resection	2 (6)
Others	12 (36.5)
**Reconstruction type**	
Oesophagogastrostomy	2 (6)
Gastrojejunostomy	1 (3.1)
No reconstruction	30 (90.9)
**Site of reconstruction**	
Intrathoracic	3 (6.1)
Abdominal	6 (18.2)
Cervical	1 (3.1)
No reconstruction	23 (72.6)

### Surgical procedures and leakage characteristics for oesophageal AL and perforations

Oesophagectomy (50 per cent) was the most frequently performed surgery requiring E-VAC therapy, followed by endoscopic procedures (14.7 per cent), gastrectomy (12.7 per cent), and bariatric operations such as gastric sleeve resection (4.9 per cent) and gastric bypasses (4.9 per cent). In patients who underwent oesophageal resection, oesophagogastrostomy was performed in all patients. The site of reconstruction was localized in the thoracic, abdominal, and cervical region in 51 per cent, 20.6 per cent and 1 per cent of the patients respectively.

No reconstruction was performed in 34 patients (33.3 per cent), 4 of whom are recorded as AL. Three patients had a staple line leak from sleeve gastrectomy and one patient had an AL following an endoscopic dilatation of an anastomotic stenosis (*Table 2*).

Oesophageal defects were divided into three subsets based on the diameter and depth of the defect. A defect diameter/depth of 0–1 cm was described as small, a diameter/depth of 1–4 cm was described as medium, and a defect more than 4 cm was described as large. In the AL group, 62.35 per cent of patients presented with a defect of 1–4 cm, 26.1 per cent had a small defect and 11.6 per cent had a large defect. In the perforation group, 51.5 per cent had a small defect, 42.4 per cent had a medium defect, and 6 per cent had a large defect.

In the AL group the defect depth was small in 17.4 per cent of cases, medium in 47.8 per cent, and large in 34.8 per cent. In the perforation group the defect depth was small in 24.2 per cent of cases, medium in 45.4 per cent, and large in 30.3 per cent.

### E-VAC therapy of the AL

In the AL group most patients (92.8 per cent) were treated within 24 h after diagnosis. After initial surgery the treatment started after a mean of 24 days (1–60 days) in the AL group. A granulated cavity was observed in 91 per cent of patients. The mean of the entire E-VAC treatment was 24.9 days (1–99 days). The placement of the Eso-Sponge® was 31 cm from the dental arch (15–50 cm, s.d. 8.3 cm). In 47.8 per cent of the AL population the Eso-Sponge® had an intraluminal and intracavitary placement. An intraluminal application on its own was performed in 20.3 per cent of cases and an intracavitary placement alone was reported in 31.9 per cent. The mean count of used Eso-Sponges® was 7.7 (1–32 sponges, s.d. 6 sponges). A mean of 7 sponge changes was reported (0–32 changes, s.d. 5.5 changes) with a mean interval of 3.1 days (2–7 days, s.d. 0.6 days). In three cases, E-VAC treatment was less than 3 days (one patient, 1 day and two patients, 2 days). In these cases, treatment with Eso-Sponge® was prophylactic for high inflammatory markers without a clearly visible oesophageal leak. A total of 75.4 per cent of the patients were treated in an ICU for the mean length of 19 days (1–141 days, s.d. 28.7 days). Additional thoracic drainage was necessary in 52 per cent of the patients. The incidence of jejunal enteral, trilumina probe (TLP) enteral, parental, and percutaneous endoscopic gastrostomy (PEG) enteral feeding was 68.1 per cent, 24.6 per cent, 4.4 per cent, and 1.5 per cent respectively.

A newly diagnosed abscess (4 per cent), fistula (9 per cent), or bleeding (6 per cent) was considered as a local complication. Systemic complications such as peritonitis (1.5 per cent), pneumonia (13 per cent), mediastinitis (13 per cent), pleural empyema (7.3 per cent), sepsis (10.1 per cent), renal failure (7.3 per cent), acute respiratory distress syndrome (4.4 per cent), or a mediastinal emphysema (5.8 per cent) were reported. A complication requiring intubation of the patient occurred in 3.9 per cent of the total cohort. Postinterventional stenosis was seen in 11.6 per cent of the patients and an additional stent was applied in 8.7 per cent. A dysphagia score of 0 was recorded for most of the patients (78 per cent), followed by a dysphagia score of 1 (14.5 per cent) and a score of 2 (6 per cent). There was only one patient (1.5 per cent) in the AL group with a dysphagia score of 3.

A reoperation was necessary in a total of 18 patients (26.1 per cent). Four reinterventions (3.9 per cent) were recorded with a definite relationship (*n* = 1) or with a suspected relationship (*n* = 3) caused by the Eso-Sponge®. The case in which a causal relationship was recorded involved an explorative laparoscopy to rule out a free perforation of the AL cavity. In two cases of the suspected causal relationship, a bronchial fistula was the reason for an operation and the third reason was a haemothorax. In the patient with a reoperation due to haemothorax, a bleeding intercostal vein was identified, which was worsened by anticoagulants. For the remaining reoperations in six cases the anastomosis had to be revised, in four cases a wound-healing disorder was diagnosed, in two cases a lavage was performed after a leakage had occurred, and in one patient each a splenic haemorrhage and a repositioning of a small bowel feeding tube were the reasons for the new operation. A clear definition of the assessment of the adverse event classification is listed when entering the events in the web-based portal (*Table S1*).

The overall mortality rate in the AL group was 6 per cent (*n* = 4 patients), and mainly due to the severe underlying disease and co-morbidities. Two patients died because of multiple organ failure. One patient died because of a malignant pleural effusion, and in another patient heart failure combined with a mediastinitis was the cause of death. All data are shown in *Tables 4* and *5*.

**Table 4.** Treatment of the endoluminal vacuum therapy

	Total *n* (%)	AL *n* (%)	Perforation *n* (%)
	102 (100)	69 (67.6)	33 (33.4)
**Treatment started within 24 h**			
Yes	92 (90.2)	65.3 (92.8)	28 (84.9)
No	10 (8.9)	5 (7.2)	5 (15.1)
**Treatment type**			
Intraluminal and intracavitary	50 (49)	33 (47.8)	17 (51.5)
Intracavitary	27 (26.5)	22 (31.9)	5 (15.2)
Intraluminal	25 (24.5)	14 (20.3)	11 (33.3)
**Intensive care unit**			
Yes	74 (72.6)	52 (75.4)	22 (66.7)
No	28 (27.5)	17 (24.6)	11 (33.3)
**Additional thoracic/pleural drainage**			
None	55 (53.9)	33 (47.8)	22 (66.7)
Thoracic drainage	41 (40.2)	34 (49.3)	7 (21.2)
Thoracic drainage lavage	6 (5.9)	2 (2.9)	4 (12.2)
**Feeding type**			
Jejunal/enteral	49 (48)	47 (68.1)	2 (6.1)
Enteral feeding tube/enteral	28 (27.5)	17 (24.6)	11 (33.3)
Parenteral	20 (19.6)	3 (4.4)	17 (51.5)
PEG/enteral	3 (2.9)	1 (1.5)	2 (6.1)
**Dysphagia score**			
0, no dysphagia: able to eat normal diet	77 (75.5)	54 (78.3)	23 (69.7)
1, moderate passage: able to eat some solid foods	18 (17.7)	10 (14.5)	8 (24.2)
2, poor passage: able to eat semi-solid foods	6 (5.9)	4 (5.8)	2 (6.1)
3, very poor passage: able to swallow liquids only	1 (1)	1 (1.5)	n.a.

AL, anastomotic leakage; PEG, percutaneous endoscopic gastrostomy.

**Table 5.** Outcome of the endoluminal vacuum therapy

	Total *n* (%)		AL *n* (%)		Perforation *n* (%)	
	102 (100)		69 (67.6)		33 (33.4)	
	Yes	No	Yes	No	Yes	No
**Granulated cavity**	88 (86.3)	14 (13.7)	63 (91.3)	6 (8.7)	25 (75.8)	8 (24.2)
**New abscess**	10 (9.8)	92 (90.2)	3 (4.4)	66 (95.6)	7 (21.2)	26 (78.8)
**Fistula**	7 (6.9)	95 (93.1)	6 (8.7)	63 (91.3)	1 (3)	32 (97)
**Bleeding, transfusion needed**	5 (4.9)	97 (95.1)	4 (5.8)	65 (94.2)	1 (3)	32 (97)
**Peritonitis**	4 (3.9)	98 (96.1)	1 (1.5)	68 (98.5)	3 (9.1)	30 (91)
**Pneumonia**	11 (10.8)	91 (89.2)	9 (13)	59 (87)	2 (6.1)	31 (94)
**Mediastinitis**	10 (10)	92 (90.2)	9 (13)	60 (87)	1 (3)	32 (97)
**Pleural empyema**	6 (5.9)	96 (94.1)	5 (7.3)	64 (92.8)	1 (3.1)	32 (97)
**Sepsis**	10 (9.8)	92 (90.2)	7 (10.1)	62 (89.9)	3 (9.1)	30 (90.9)
**Renal failure**	5 (4.9)	97 (95.1)	5 (7.3)	64 (92.8)	n.a.	33 (100)
**ARDS**	3 (2.9)	99 (97.1)	3 (4.4)	65 (94.2)	n.a.	33 (100)
**Mediastinal emphysema**	5 (4.9)	97 (95.1)	4 (5.8)	65 (94.2)	1 (3.1)	32 (97)
**Intubation**	4 (3.9)	98 (96.1)	2 (2.9)	67 (97.1)	2 (6.1)	31 (93.9)
**Stenosis**	10 (9.8)	92 (90.2)	8 (11.6)	61 (88.4)	2 (6.1)	31 (93.9)
**Stent applied**	9 (8.8)	93 (91.2)	6 (8.7)	63 (91.3)	3 (9.1)	30 (90.9)
**Reoperation**	26 (25.5)	76 (74.5)	18 (26.1)	51 (73.9)	8 (12.1)	25 (75.8)
**Death**	7 (6.9)	95 (93.1)	4 (5.8)	65 (94.2)	3 (9)	30 (91)
	Mean (s.d.)		Mean (range)		Mean (range)	
**Therapy duration (days)**	26.41 (24.68)		24.95 (1–99)		30.09 (2–141)	
**Distance from the arch (cm)**	30.23 (9.22)		30.91 (15–50)		28.82 (15–45)	
**Defect diameter (cm)**	2.54 (2.16)		2.77 (0–15)		2.04 (0–8)	
**Defect depth (cm)**	4.49 (4.20)		4.33 (0–15)		4.8 (0–24)	

AL, anastomotic leakage; ARDS, acute respiratory distress syndrome.

### E-VAC therapy of the perforations

Most patients in the perforation group were treated within the first 24 h (84.9 per cent). A sealed and granulated cavity was observed in 75.76 per cent of the treated patients. The mean of E-VAC treatment was 30.1 days (2–141 days, s.d. 32.8 days) in the perforation group.

The mean distance from the dental arch was 30 cm (15–45 cm, s.d. 10.9 cm). In 51.6 per cent of the cases, the Eso-Sponge® had an intraluminal and intracavitary placement. A mean of 9 Eso-Sponges® were used (1–37 sponges, s.d. 9.9 sponges) and changed a mean of 7.4 times (0–36 changes, s.d. 8.6 changes) with a change interval of 3 days (2–4 days, s.d. 0.5 days). A total of 66.7 per cent of the patients had to be treated in an ICU for a mean length of 22 days (1–114 days, s.d. 30.2 days). Additional thoracic drainage was necessary in 33 per cent of the patients. The incidence of jejunal enteral, TLP enteral, parental, and PEG enteral feeding was 6.1 per cent, 33.3 per cent, 51.5 per cent, and 6.1 per cent respectively.

A new abscess (21 per cent), fistula (3 per cent), or bleeding (3 per cent) was recorded within the group. Systemic complications such as peritonitis (9 per cent), pneumonia (6 per cent), mediastinitis (3 per cent), pleural empyema (3 per cent), sepsis (9 per cent), or mediastinal emphysema (3 per cent) were recorded. Procedural complications included bleeding (3 per cent), aspiration (3 per cent), and intubation (6 per cent). A stenosis was seen in 6 per cent of patients and an additional stent was applied in 9 per cent.

A dysphagia score of 0 was recorded for most of the patients (69.7 per cent), followed by dysphagia score of 1 (24 per cent), and a score of 2 (6 per cent).

A reoperation was needed in eight patients. In five cases an oesophageal resection had to be performed due to a persistent wound cavity at the site of the perforation. In three of the eight cases a potential causal relationship between the Eso-Sponge® and the reoperation was found. One operation was carried out to perform an abdominal lavage, one because of a mediastinal abscess, and the case with a possible causal relationship to Eso-Sponge® was a thoracotomy for bleeding.

The mortality rate was 9 per cent (*n* = 3 patients), mainly due to the underlying disease and co-morbidities of the patients. One patient died due to a myocardial infarction, one due to intraoperative pulmonary bleeding, and the third due to a fulminant pulmonary embolism. All data are shown in *Tables 4* and *5*.

### Influencing variables on the Eso-Sponge® therapy success

Variables that might influence the outcome of the Eso-Sponge® treatment, and therefore the condition of the defect, were evaluated. The presence of a granulated cavity in the defect was considered a positive response. The start of therapy within 24 h (*P* = 0.07), previous neoadjuvant therapy (*P* = 0.09), and presence of pneumonia (*P* = 0.06) were not predictive.

The presence of mediastinitis was associated with significant failure of EVT (*P* = 0.002). EVT applied in patients with an AL had better outcomes than for oesophageal perforations (*P* = 0.026). In particular, the height from the dental arch of the leakage or perforation was identified as a highly significant risk factor (*P* = 0.0088). A success rate of 89 per cent was found for a distance of more than 20 cm.

## Discussion

Oesophageal perforations and AL following oesophageal resections can lead to serious complications, resulting in high mortality^7,19^. Historically the only options for treatment were stents or surgical treatment^20^. A few years ago, EVT was established as an endoscopic option^14^ with potential advantages and reduced mortality compared with previous treatments^14^. Contrary to previous single-centre studies, a standardized protocol in a multicentre setting was used, which allows wider applicability of data concerning EVT. In general, EVT has been deemed a reliable and effective minimally invasive procedure^21–24^. It is well tolerated and at the same time the complication rates are relatively low^25,26^.

This study demonstrated a success rate of 91 per cent for EVT in patients, consistent with the literature. Loske *et al*. reported success rates of 60–100 per cent in a recent review of the available literature on endoscopic negative pressure therapy in the upper GIT^27^.

There was a low complication rate with stenosis in 10 per cent, comparable to previous studies^28^. Serious complications have rarely been described to date and include bleeding due to vascular erosion and the development of an oesophagobronchial fistula^8,29^. In this study seven patients developed a fistula after EVT. This is consistent with a previous study that demonstrated oesophagobronchial fistula in 2 out of 35 patients^30^.

The reoperation rate after EVT treatment was high at 26 per cent compared with the data of Brangewitz *et al*.^13^, where only one revisional surgery was performed on 32 patients. However, in another study the rate was four out of nine patients^31^. This demonstrates the challenging nature of this type of intervention in small single-centre studies to be able to accurately compare outcomes. The majority of reoperations in this study were due to secondary problems (such as wound infection, percutaneous endoscopic jejunostomy dislocation, and haemothorax). Only 9 of the 26 reoperations were performed for anastomosis revision or treatment of surgical perforation.

The mortality rate was 6 per cent, which was similar to published data (0–18 per cent)^7,32,33^. Further stratifying of patients into subgroups with AL and perforation, mortality rates were 6 per cent and 9 per cent respectively. These results seem favourable for Eso-Sponge® therapy, considering Schorsch *et al*. published a mortality rate for AL of 12–35 per cent and a perforation-related mortality rate of 11.7 per cent^30^.

Generally, the optimal time point for EVT is thought to be immediately after the diagnosis of an oesophageal AL. It improves clinical outcome in comparison with a postponed treatment, more than 24 h after detection, which is associated with a prolonged duration of hospital stay and a higher mortality rate^21,25^. In our study, commencement of EVT within 24 h could be achieved in 84 per cent of the perforation cohort and even in 91 per cent of the AL cohort. This factor did not, however, influence outcomes.

EVT can be safely and effectively used in patients who have been previously treated with chemoradiotherapy and those presenting with severe leaks. Several studies describe poor wound healing and AL rates after neoadjuvant chemotherapy^34,35^. Contrary to the findings of Min *et al*., no significant influence of neoadjuvant pretreatment on EVT was identified in this study^36^.

In this study only mediastinitis had a significant influence on the healing rate during EVT. The incidence of mediastinitis was low at 9.8 per cent of the overall cohort compared with previous studies with 43 per cent^24^. The lower healing rate for those patients could be explained by a delayed leak drainage and thus prevention of further contamination, which is the underlining concept of therapy with EVT^37^.

In our group of patients, sponges were changed at an interval of 3–4 days, which has proven effective in our clinical experience. It gives the sponge sufficient time to induce granulation of the wound cavity and yet still ensures easy removal. This finding is in keeping with the current literature^16,24,28^, although there are promising studies reporting an alternating cycle of 1–2 weeks^38^.

While the results of this study are promising, there are limitations. The major limitation of our study is the lack of a comparative cohort with alternative therapies (such as stent therapy). In the present study, however, no comparative analysis of the results was intended, but rather a descriptive characteristic of the present procedure in a uniquely large patient cohort, applying a highly standardized application of treatment. This important aspect is missing in the current literature^39^.

The lack of standardization of clinical conditions can directly affect the results. Data about fistula size, laboratory studies, sepsis, antibiotic regimen, or an objective assessment through a predictive score system (such as Acute Physiology and Chronic Health Evaluation score) could reduce the risk of bias. However, this study is a presentation of the EVT within the framework of a uniform standardized procedure.

This multicentre study confirms the promising results of standardized EVT for the management of upper gastrointestinal ALs and perforation.

## Supplementary Material

zrac030_Supplementary_Data

## Data Availability

The data that support the findings of this study are available from the sponsor of this study upon reasonable request.
