# Supplementary material for: Eso-Sponge® for anastomotic leakage after oesophageal resection or perforation: outcomes from a national, prospective multicentre registry
Source: BJS Open. 2022 Apr 22;6(2):zrac030. doi: 10.1093/bjsopen/zrac030 (PMC9023777; doi:10.1093/bjsopen/zrac030)
Supplement: zrac030_Supplementary_Data [file zrac030_supplementary_data.zip › Supplementary_Figure_1.docx]

**Demographic data**

**Gender**
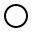
 male
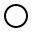
 female

**Age**

years

**Weight**

kg

**Height**

cm

**BMI**

(calculated by the system)

**General healthy status**

According to ASA the patient is classified:


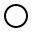
 ASA 1 - normal healthy patient


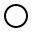
 ASA 2 - patient with mild systemic disease


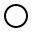
 ASA 3 - patient with severe systemic disease


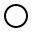
 ASA 4 - patient with severe systemic disease that is a constant threat to life **Risk factors**
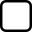
 none


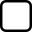
 Diabetes - not insulin-dependent


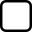
 Diabetes - insulin-dependent


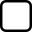
 Peripheral arterial disease (PAVK)


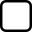
 Heart insufficiency


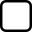
 Immunosuppression


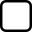
 Renal insufficiency without dialysis


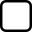
 Renal insufficiency with dialysis


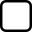
 Liver structure defect


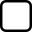
 Smoking


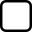
 Alcohol consumption

**Main diagnosis**


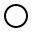


Esophageal cancer - upper


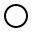


Esophageal cancer - middle


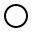


Esophageal cancer - lower


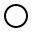


Cancer of the gastric cardia


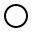


Cancer of the gastric corpus


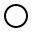


Cancer of the gastric antrum


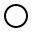


Obesity


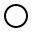


Boerhaave's Syndrome


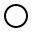


Iatrogenic perforation


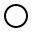


Bole related perforation


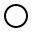


Other, please specify:

X

**Histology performed**


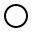


not done


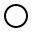


squamous cell cancer


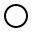


adenocarcinoma


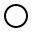


other, please specify:

X

**Treatment prior to surgery**


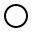


none


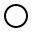


Chemotherapy


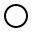


Radiotherapy


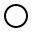


Radiochemotherapy


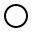


Other, please specify:

X

**Type of Resection/Procedure**


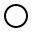


total esophagectomy


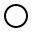


partial esophagectomy


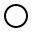


total gastrectomy


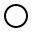


partial gastrectomy


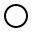


gastric sleeve resection


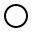


gastric bypass


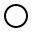


fundoplication


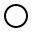


endoscopic procedure


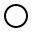


other, please specify:

X

**__comment:**

X

**Type of reconstruction**


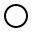


no reconstruction


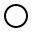


esophagogastrostomy


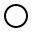


esophagocolostomy


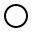


esophagojejunostomie


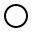


gastrojejunostomy


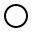


other, please specify:

X

**In case of reconstruction: Site of anastomosis/stapler/suture line**


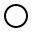


cervical


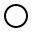


intrathoracic


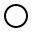


abdominal


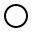


other, please specify:

X

**Cause of leakage:**


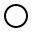


Anastomotic insufficiency


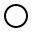


Perforation cervical


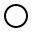


Perforation thoracic


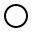


Perforation abdominal


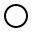


Other, please specify:

X

**Date of resection / surgery / procedure**

dd.mm.yyyy X

**Date of perforation**

dd.mm.yyyy X **When was the leakage diagnosed? (days postoperatively)**

days_

**Date of Eso-Sponge treatment start**

dd.mm.yyyy X

**Was the treatment started within 24h after the diagnosis of the leakage/perforation?**


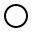


yes


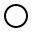


no, please specify when and why

X

**Leakage Characteristics**

**Distance from the arch**

cm

**Maximum size of circumference dehiscence**


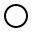
 1/4


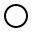
 1/2


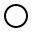
 3/4


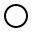
 4/4

**Maximum size of the defect - depth**

cm

**Maximum size of the defect - diameter**

cm

**Treatment type**


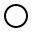


intracavitary


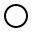


intraluminal


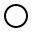


intraluminal and intracavitary


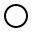


other, please specify

X

**Treatment drainage**


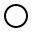


transnasal


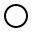


transoral


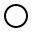


transnasal and transoral


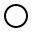


other, please specify

X

**Patient has been in the intensive care unit**


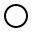


no


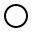


yes, total days in the ICU:

days_

**Patient under ventilator treatment**


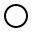


no


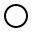


yes, total days on mechanical ventilation:

days_

**Additional application of a thoracic / pleural drainage**


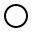
 None


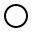
 Bülau drainage


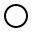
 Bülau drainage lavage

**Feeding type**
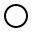
 parenteral


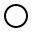
 TLS - enteral


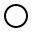
 PEG - enteral


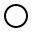
 Jejunal - enteral


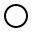
 Other

**Duration of feeding:**

days_

**VAC Sponge Treatment**

**Number of sponge changes**

X

| **Number of total used sponges**  X  **Average Replacement intervals**  days_  **Date of treatment end**  dd.mm.yyyy | X |
| --- | --- |

**Local Outcome**

**Granulated cavity**
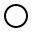
 no
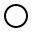
 yes

**If yes, size of the cavity diameter**

cm

**If yes, size of cavity depth**

cm

**Stent**


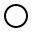


no


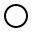


yes, please specify:

X

**Stenosis**


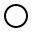


no


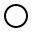


yes, size of diameter:

mm

**Functional Outcome**

**Dysphagia score**

Score

1. - no dysphagia: able to eat normal diet
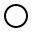

2. - moderate passage: able to eat some solid foods
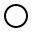

3. - poor passage: able to eat semo-solid foods
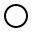

4. - very poor passage: able to swallow liquids only
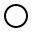

5. - no passage: unable to swallow anything
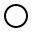


**Re-operation**


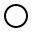


no


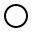


yes, date:

dd.mm.yyyy

**If Re-operation yes, please specify reason and procedure:**

Lavage

Reconstruction of the anastomosis/resuturing

Discontinuing resection

Abscess discharge - abdominal

Abscess discharge - thoracic

Closure with flap of muscle

Bronchial fistula

Other, please specify:

X

**Death**

no

yes, date:

dd.mm.yyyy

**If death yes, please specify cause of death:**

**In case of re-operation or death please fill in an Adverse Event form.**

**Complications**  no

yes, please fill in an Adverse Event Form and specify below:

**Local complications**

Date of occurance (DD.MM.YYYY)

Date of occurance

New AbscessX

FistulaX

Bleeding, transfusion neededX

| Eso-Sponge Registry Study No.: AAG-O-H1317 | **Discharge/Complications** | Study Center No.:  Patient No.  Page | 12 of 15 |
| --- | --- | --- | --- |

OtherX

| **Systemic complications** | Date of occurance (DD.MM.YYYY)  Date of occurance |
| --- | --- |

New PeritonitisX

New PneumoniaX

MediastinitisX

PleuraempyemaX

SepsisX

Renal failureX

ARDSX

Mediastinal emphysemaX

OtherX **Procedure complications**

Date of occurance (DD.MM.YYYY)

Date of occurance

BleedingX

AspirationX

IntubationX

| Eso-Sponge Registry Study No.: AAG-O-H1317 | **Discharge/Complications** | Study Center No.:  Patient No.  Page | 13 of 15 |
| --- | --- | --- | --- |

OtherX

**Date of discharge**

dd.mm.yyyy X

**In the case of local, systemic or procedure complications please fill in an Adverse Event form.**

**1.0 Adverse Event no.**

| Eso-Sponge Registry Study No.: AAG-O-H1317 | **Adverse Event (AE)** | Study Center No.:  Patient No.  Page | 14 of 15 |
| --- | --- | --- | --- |

X

**1.1**

**Description of the event / Symptoms**

**1.2 Onset date**

dd.mm.yyyy X

**1.3 Finish date**

dd.mm.yyyy X

**1.4**

**Causal relationship to the investigated product**

no

yes, please specify below

possible, please specify below

Comment:

X

**1.5 Is the adverse event a serious adverse event?**

(AEs leading to death, life-threatening conditions, permanent serious impairments, prolonging hospital stay, demanding surgical intervention or fetus injuries)

no yes

| Eso-Sponge Registry Study No.: AAG-O-H1317 | **Adverse Event (AE)** | Study Center No.:  Patient No.  Page | 15 of 15 |
| --- | --- | --- | --- |

X

**1.6**

**Therapeutic measures**

none

non-invasive (medication-name, dosis, application, start/end, physiotherapy etc.)

Revision/Reoperation, please specify date and explain procedure performed

Other, please specify

X

X

**1.7 Outcome**  resolved, no sequelae resolved, with sequelae ongoing death unknown
