# Supplementary material for: Eso-Sponge® for anastomotic leakage after oesophageal resection or perforation: outcomes from a national, prospective multicentre registry
Source: BJS Open. 2022 Apr 22;6(2):zrac030. doi: 10.1093/bjsopen/zrac030 (PMC9023777; doi:10.1093/bjsopen/zrac030)
Supplement: zrac030_Supplementary_Data [file zrac030_supplementary_data.zip › Supplementary_Table_1.docx]

**Table S1: Definition of Adverse Event causality**

| **Causality code** | **Definition** |
| --- | --- |
| Not assessable | A report suggesting an Adverse Event, which cannot be judged because information is insufficient or contradictory, and which cannot be supplemented or verified. |
| Unlikely | A clinical event, including laboratory test abnormality, with a temporal relationship, which makes a causal relationship improbable, and in which other drugs / treatments, chemicals or underlying disease(s) provide plausible explanations. |
| Possible | A clinical event, including laboratory test abnormality, with a reasonable temporal relationship to administration of the drug / treatment, but which also could be explained by concomitant diseases or other drugs / treatments or chemicals. |
| Probable | A clinical event, including laboratory test abnormality, with a reasonable temporal relationship to administration of the drug / treatment, unlikely to be attributable to concomitant disease(s) or other drugs / treatments or chemicals, and which follows a clinically reasonable response on withdrawal (dechallenge). Rechallenge information is not required to fulfil this definition. |
| Certain | A clinical event, including laboratory test abnormality, occurring in a plausible time relationship to study treatment and which cannot be explained by concomitant disease(s), other drugs / treatments or chemicals. The response to withdrawal of the treatment (dechallenge) should be clinically plausible. The event must be unambiguously either pharmacologically or as phenomenon, using in satisfactory rechallenge procedures if necessary. |
